# Metal behavior in the extremes of dynamics

**DOI:** 10.1038/s41598-018-23566-1

**Published:** 2018-03-26

**Authors:** Aleksander Zubelewicz

**Affiliations:** University of New Mexico, Albuquerque, NM USA

## Abstract

When the rate of loading is faster than the rate at which material absorbs and converts energy to plastic work and damages, then there is an excess of energy that is partly stored in the material’s microstructure and the rest of it triggers micro-dynamic excitations. The additional storage necessitates the development of plastic flow constraints and is directly responsible for the observed dynamic strengthening. At extreme conditions, we find that the micro-excitations contribute to the dynamic behavior. The phenomena are universally observed in metals, frictional materials and polymers. In essence, strong dynamics creates conditions at which materials are pushed from equilibrium and temporarily reside in an excited state of behavior. This study is focused on the behavior of metals. The concept is incorporated into a mechanisms-based constitutive model and is examined for annealed OFHC copper.

## Introduction

When energy is delivered to a material with rates that are faster than the rate at which the material converts the excess energy to plastic work, then the uncompensated energy is partly stored in the material, while the rest of it is converted to micro-dynamic excitations. In metals, the observed strengthening mechanism^1^ is linked to kinetics of the drag-controlled dislocation glide under applied stress^2,3^. Often, it is assumed^4^ that the thermally activated dislocation mechanism operates at all strain rates, while others^5^ have argued that both thermal activation and drag-controlled mechanisms coexist at high strain rates. A comprehensive review of the theoretical concepts and models is presented in refs^6,7^. In various constitutive descriptions, the effort is focused on connecting the material responses at high strain rates with the rapid increase of dislocation density and the development of fine dislocation structures. Subsequently, the microstructural evolution is coupled with external stimuli such as strain rate and temperature.

Experimental observations presented in ref.^8^ suggest that the dynamic behaviors arise due to the intrinsic resistance of lattice to motion of dislocations. This mechanism competes with an extrinsic resistance exerted by defects such as vacancies, interstitials and dislocations. The phenomena are studied with the use of standard split-Hopkinson pressure bar and Taylor cylinder tests^9^, where the achievable strain rates are in the range of 10^4^/*s*. The experiments have been further modified for a combined pressure-shear loading^8^, and then, the strain rates can reach a range of 10^5^/*s* to nearly 10^7^/*s*. As reported, the strain rates produce thermal instabilities and the fraction of plastic work converted to heat is much lower from the common ratio of β = 0.9. Even stronger loading is achieved in gas gun experiments, where energy is delivered in much shorter times^10^. The micro-dynamic excitations are consistently detected in acoustic emission (AE) measurements. In metals and rocks, the rate of AE counts is proportional to the rate of plastic strain^11,12^, where in metals bursts of acoustic events are linked to the dislocation activities. Lifespan of the excitations is short and is measured in the range of microseconds. We conjecture that extreme strain rates exceeding 10^4^/*s* produce conditions where the micro-kinetic energy explicitly contributes to the dynamic behavior. The word “extreme” refers to the conditions.

Since dynamic strengthening and micro-kinetic excitations have been observed in metals, rocks and polymers, we suggest that the dynamic phenomena follow essentially similar processes in all the materials. We narrow the study and focus our considerations on the behavior of metals. The objective is to explain the existence of dynamic overstress and micro-kinetic excitations in metals subjected to extreme loading. During fast processes, conversion of energy to plastic work can be delayed, and then, the energy is temporarily stored in the material and triggers dynamic excitations. In comparison with quasi-static processes, strong dynamic stimuli push the material further away from the near equilibrium behavior. At these conditions, the material adjusts its dislocation arrangement and dissipates large amounts of energy. The dynamic responses are incorporated into our constitutive model and are studied under a broad range of strain rates and temperatures.

## Results

**Figure 1:**
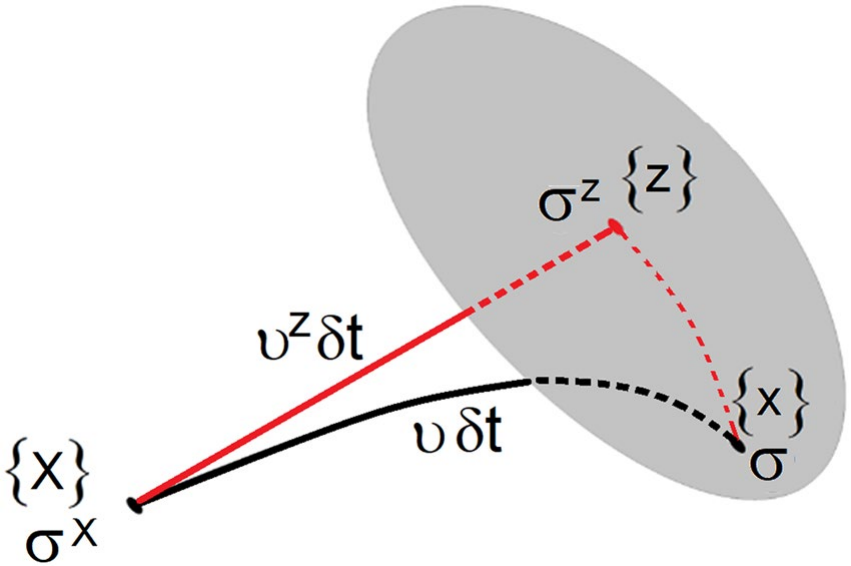
Pictorial representation of stress perturbations. Stresses are projected onto a surface normal to the direction of particle velocity. The perturbed process affects stresses and is responsible for the activation of dynamic excitations.

A transition from one near equilibrium state to another triggers small spatial-temporal perturbations. During slow processes, the local instabilities are negligibly small. A different scenario arises at strong dynamics (strain rates above 10^4^/*s*), where the near equilibrium behaviors cannot be preserved. We replicate the process by monitoring trajectory of a selected material particle and its surroundings, Fig. 1. Initially, the particle occupies position {***X***} and the stress is **σ**^*X*^. Short time later δ*t*, the particle moves to the next available position {***x***} and the stress becomes **σ**. The actual path may deviate from the near equilibrium trajectory, which would place the particle in position {**z**} with the stress **σ**^*Z*^. The requirement here is that the equations of motion $\nabla \cdot \,{\boldsymbol{\sigma }}=\rho \dot{{\boldsymbol{\upsilon }}}$ are satisfied in all scenarios, where the particle acceleration is $\dot{{\boldsymbol{\upsilon }}}$ and mass density is *ρ*. Deviation from the near equilibrium pathway perturbs stresses. We evaluate the perturbations by calculating stress tractions on the surface $\,\partial {V}_{0}$ normal to the direction of the particle velocity ***n***. Tractions in {**z**} and {***x***} positions may be different $({{\boldsymbol{\sigma }}}^{z}\cdot {\boldsymbol{n}}\ne {\boldsymbol{\sigma }}\cdot {\boldsymbol{n}})$ and the difference is responsible for stress perturbations so that

1 $$\delta {\boldsymbol{\sigma }}=\frac{{l}_{c}}{{V}_{0}}{\int }_{\partial {V}_{0}}({\boldsymbol{\sigma }}-{{\boldsymbol{\sigma }}}^{z})\cdot ({\boldsymbol{n}}\otimes {\boldsymbol{n}})dS\,$$

The perturbations are stretched over a spatial domain defined by the length $\,{l}_{c}$, where size of the domain varies during active loading process. We use divergence theorem and reduce volume $\,{V}_{0}$ to a material point, and then, the stress perturbations become $\delta {\boldsymbol{\sigma }}=\rho {l}_{c}\,(\delta \dot{{\boldsymbol{\upsilon }}}\otimes {\boldsymbol{n}}+{\boldsymbol{n}}\otimes \delta \dot{{\boldsymbol{\upsilon }}})/2$. The difference in the particle acceleration is $\,\delta \dot{{\boldsymbol{\upsilon }}}=(\dot{{\boldsymbol{\upsilon }}}-{\dot{{\boldsymbol{\upsilon }}}}^{z})$ and $\,{{\boldsymbol{\upsilon }}}^{z}$ is particle velocity along the original trajectory. We introduce symmetric momentum tensor $\,{\boldsymbol{\psi }}=\rho (\delta {\boldsymbol{\upsilon }}\otimes {\boldsymbol{n}}+{\boldsymbol{n}}\otimes \delta {\boldsymbol{\upsilon }})/2$ and express the stress perturbations $\delta {\boldsymbol{\sigma }}={l}_{c}\dot{{\boldsymbol{\psi }}}$ accordingly.

At the prescribed rate of total strain ${\dot{{\boldsymbol{H}}}}^{t}$, a part of stress ${\boldsymbol{C}}\cdot {\dot{{\boldsymbol{H}}}}^{t}$ cannot be fully accommodated by lattice. In such a circumstance, the stress excess excites material particles $\dot{{\boldsymbol{\psi }}}/\rho$ and it does so in the $\,{l}_{c}$-domain only. The rate of stress is affected by the excitations $\kappa \dot{{\boldsymbol{\psi }}}/\rho {l}_{c}$, hence

2 $$\dot{{\boldsymbol{\sigma }}}+\frac{\kappa }{\rho {l}_{c}}\dot{{\boldsymbol{\psi }}}={\boldsymbol{C}}\cdot {\dot{{\boldsymbol{H}}}}^{t}.$$

In this expression, the forth order elastic tensor ${\boldsymbol{C}}=\lambda 1\otimes 1+2\,\mu {\boldsymbol{I}}$ is defined in terms of Lame constant *λ* and shear modulus *μ*, where $\lambda =B-2\mu /3$ and *B* is bulk modulus, **1** is Kronecker delta and ***I*** is fourth order identity tensor. We emphasize that the expression (2) is also valid in an elastically anisotropic material. At quasi-static conditions, the vanishing accelerations $(\dot{{\boldsymbol{\psi }}}/\rho \to 0)$ act in a vanishing domain $\,{l}_{c}$. Therefore, the micro-excitations $\,\dot{{\boldsymbol{\psi }}}$ activate the viscous overstress $\kappa \dot{{\boldsymbol{\psi }}}/\rho {l}_{c}$ at all strain rates, where at slow rates we have $\kappa \dot{{\boldsymbol{\psi }}}/\rho {l}_{c}\to {\boldsymbol{C}}\cdot {\dot{{\boldsymbol{H}}}}^{p}$. The rate of plastic strain is denoted as ${\dot{{\boldsymbol{H}}}}^{p}$. The resistance to plastic flow $\,\kappa$ is sensitive to temperature $\,T$ and is given in units of viscosity $\kappa ={A}_{D}(T)\,{\kappa }_{0}$, where *κ*_0_ is a material constant and *A*_*D*_ is a factor that describes thermal resistance to plastic flow. This factor is derived in Methods. In metals, the lattice relaxation time is very short and, for this reason, the elastic relations are assumed to be preserved at all times

3 $$\dot{{\boldsymbol{\sigma }}}={\boldsymbol{C}}\cdot {\dot{{\boldsymbol{H}}}}^{e},$$

where the rate of elastic strain is ${\dot{{\boldsymbol{H}}}}^{e}$. While the viscous stress initiates and mobilizes dislocations, the stress perturbations *δ****σ*** slow down plastic flow and, therefore, act as a drag on dislocations

4 $${\dot{{\boldsymbol{H}}}}^{t}={\dot{{\boldsymbol{H}}}}^{e}+({\dot{{\boldsymbol{H}}}}^{p}-\frac{\delta {\boldsymbol{\sigma }}}{\kappa }).$$

We eliminate the momentum tensor by combining (2), (3) and (4) and obtain ${\dot{{\boldsymbol{H}}}}^{p}={\boldsymbol{P}}\cdot ({\dot{{\boldsymbol{H}}}}^{t}-{\dot{{\boldsymbol{H}}}}^{e})$, where the forth order drag tensor ${\boldsymbol{P}}={\boldsymbol{I}}+{R}_{k}^{2}{\boldsymbol{C}}/\mu$ slows down plastic flow. The rate of total strain becomes ${\dot{{\boldsymbol{H}}}}^{t}={\dot{{\boldsymbol{H}}}}^{e}+{{\boldsymbol{P}}}^{-1}\cdot {\dot{{\boldsymbol{H}}}}^{p}$. The resistance to plastic flow is *R*_*k*_ = *υ*_*c*_/*υ*_*s*_, where shear velocity is $\,{\upsilon }_{s}=\sqrt{\mu /\rho }$. The length *l*_*c*_ = *τ*_0_*υ*_*c*_ is proportional to the relaxation time *τ*_0_ = *κ*/*μ* and its size is defined by the velocity *υ*_*c*_, at which the perturbations are spreading out from the source. The relaxation time is well-defined, and the velocity is subsonic and requires calibration. We chose to calibrate the resistance to plastic flow, thus *υ*_*c*_ = *R*_*k*_*υ*_*s*_.

The drag tensor ***P*** channels plastic deformation along most favorable lattice pathways. The rate of effective plastic strain becomes

5 $${\dot{{\boldsymbol{H}}}}^{pe}={{\boldsymbol{P}}}^{-1}\cdot {\dot{{\boldsymbol{H}}}}^{p}.$$

This channeling is most pronounced in textured metals. In elastically isotropic and plastically incompressible metals, the drag tensor takes simple form and is ${{\boldsymbol{P}}}^{-1}={\boldsymbol{I}}/(1+2{R}_{k}^{2})$. The perturbations cannot propagate faster than shear velocity, thus the strongest drag occurs when ***P***^−1^ → ***I***/3. At extreme strain rates, dislocations multiply rapidly and settle in entangled configurations. In a single crystal copper subjected to laser shock^13^, molecular dynamics simulations indicate that the structures mature in tens picoseconds. The process is much slower in polycrystalline samples. It is estimated^14^ that aluminum alloys may reach equilibrium in microseconds. In the *R*_*k*_-constrained configuration, the viscous overstress is calculated from (2), (3) and (4) and is $\kappa \dot{{\boldsymbol{\psi }}}/\rho {l}_{c}={\boldsymbol{C}}\cdot ({\dot{{\boldsymbol{H}}}}^{t}-{\dot{{\boldsymbol{H}}}}^{e})$. We invoke (5) and obtain $\kappa \dot{{\boldsymbol{\psi }}}/\rho {l}_{c}={\boldsymbol{C}}\cdot {\dot{{\boldsymbol{H}}}}^{pe}$. The stress undergoes continuous relaxation and the mechanism is best described by the Maxwell process

6 $${\dot{{\boldsymbol{S}}}}^{a}+\frac{1}{2\,{\tau }_{0}}{{\boldsymbol{S}}}^{a}={\boldsymbol{C}}\cdot {\dot{{\boldsymbol{H}}}}^{pe},$$

where the active overstress is ***S***^*a*^. The change of momentum $\dot{{\boldsymbol{\psi }}}={R}_{k}\,{\boldsymbol{C}}\cdot {\dot{{\boldsymbol{H}}}}^{pe}/{\upsilon }_{s}$ undergoes relaxation too, and the rate of active momentum is $\,{\dot{{\boldsymbol{\psi }}}}^{a}={R}_{k}{\dot{{\boldsymbol{S}}}}^{a}/{\upsilon }_{s}$. Also, Eqn. 6 indicates that the excitations are dissipated over time. Next, we calculate the actual rate of micro-kinetic energy $\dot{{\mathscr{K}}}={\dot{{\boldsymbol{\psi }}}}^{a}:{{\boldsymbol{\psi }}}^{a}/\rho$. Lattice storage is calculated by multiplying both sides of (6) by ***S***^*a*^/2*μ* and, then

7 $$({\dot{W}}_{l}+\frac{1}{{\tau }_{0}}{W}_{l})={\dot{W}}_{k},$$

where ${\dot{W}}_{k}={{\boldsymbol{S}}}^{a}:{\dot{{\boldsymbol{H}}}}^{pe}$ and the rate of lattice storage is ${\dot{W}}_{l}={{\boldsymbol{S}}}^{a}:{\dot{{\boldsymbol{S}}}}^{a}/2\mu$. According to (7), remaining energy is dissipated ${\dot{D}}_{k}={W}_{l}/{\tau }_{0}$. The storage *W*_*l*_ is partially hidden in the preexisting and stress-induced heterogeneities, thus only a fraction of the energy can be measured at the scale of continuum. However, entire energy *W*_*l*_ contributes to the development of flow constraints. We introduce a non-dimensional variable *ξ*_*R*_ and use it for counting the storage events. The events *ξ*_*R*_ are stochastic and are assumed to obey the Weibull distribution. In this manner, frequency of the events is ${f}_{R}={k}_{R}\,{\xi }_{R}^{{k}_{R}-1}exp(-{\xi }_{R}^{{k}_{R}})$ and the events multiply with the rate *k*_*R*_. The constraints enable the additional storage and, therefore, we have ${R}_{k}={\int }_{0}^{{W}_{l}/{G}_{R}}{f}_{R}\,d{\xi }_{R}$, where the activation energy is *G*_*R*_. Consequently, the resistance to plastic flow is

8 $${R}_{k}=1-exp[-{({W}_{l}/{G}_{R})}^{{k}_{R}}].$$

It is expected that the constraints are partly retained after the material is brought to a thermodynamic equilibrium. The constraints arise due to the competition between the viscous overstress that activates the plastic flow and the stress perturbations that slow down the process. In fact, it has already been suggested that plastic processes result from the intrinsic and extrinsic resistance to flow, where thermal softening is in a direct competition with the lattice resistance to flow^8^. In our approach, we show that the dynamic phenomena manifest themselves not only in terms of the dynamic strengthening, but also are associated with the dynamic excitations.

### Thermodynamics interpretation

In the excited state, change of the energy excess is *δW*_*t*_ = ***S***^*a*^:*δ****H***^*p*^. Part of the energy is absorbed by the material *δW*_*k*_ = ***S***^*a*^:*δ****H***^*pe*^ and the rest generates dynamic excitations $\delta {\mathscr{K}}$. The excitations are partly dissipated $2\,{R}_{k}^{2}\,\delta {D}_{k}$, hence $\delta {\mathscr{K}}+2{R}_{k}^{2}\delta {D}_{k}={{\boldsymbol{S}}}^{a}:(\delta {{\boldsymbol{H}}}^{p}-\delta {{\boldsymbol{H}}}^{pe})$. Since $\delta {{\boldsymbol{H}}}^{p}-\delta {{\boldsymbol{H}}}^{pe}=2{R}_{k}^{2}\delta {{\boldsymbol{H}}}^{pe}$, we rewrite the expression and obtain $\delta {\mathscr{K}}=2{R}_{k}^{2}(\delta {W}_{k}-\delta {D}_{k})$. The energy excess *W*_*t*_ is included in Helmholtz free energy *F* = *U*(***H***^*t*^,*S*,*R*_*k*_) − (*ST* + *W*_*t*_), where entropy is *S*. Consequently, the change of free energy becomes

9 $$\delta F={\boldsymbol{\sigma }}:\delta {{\boldsymbol{H}}}^{t}-(S\delta T+\delta {\mathscr{K}}).$$

The constraints *R*_*k*_ enable the dynamic storage (∂*U*/∂*R*_*k*_)*δR*_*k*_ = *δW*_*l*_, while the entropic term is $S\delta T=\delta {W}^{p}+(1+2{R}_{k}^{2})\delta {D}_{k}$. At quasi-static and mild dynamic conditions, the micro-kinetic energy is negligibly small. At extreme strain rates, large excitations may destabilize the internal structure of metals such that $\delta {\mathscr{K}}\propto (\delta {\boldsymbol{C}}\cdot {{\boldsymbol{H}}}^{e}):{{\boldsymbol{H}}}^{e}/2$, where the degradation of elastic properties is defined in *δ****C***. In fact, the formation of the debris cloud under hypervelocity impacts is an observed phenomenon^15,16^.

## Discussion

The concept of dynamic excitations is incorporated into our viscoplasticity model. Details of the model are presented in Methods. Here, we provide a brief summary of the model. First, we introduce thermodynamic relevant constants given here in terms of shear and bulk moduli (*μ*,*B*), specific heat *C*_*p*_ and mass density *ρ*. Stress is calculated from $\dot{{\boldsymbol{\sigma }}}={\boldsymbol{C}}\cdot ({\dot{{\boldsymbol{H}}}}^{t}-{\dot{{\boldsymbol{H}}}}^{pe})$. The constitutive model consists of three components.

1. We describe thermal activation processes, where the derived thermal activation factor is ${A}_{D}(T)=1-exp[-{g}_{a}{(\frac{{T}_{c}}{T}-\frac{{T}_{c}}{{T}_{m}})}^{{k}_{a}}]$. In the expression, the transition of flow mechanism occurs at *T*_*c*_ and melting point is *T*_*m*_. The rate of dislocation multiplication *k*_*a*_ further quantifies thermal activation. The factor *g*_*a*_ is the activation energy multiplier.
2. The description of plastic flow is provided in the second component. The rate of plastic flow is defined as ${\dot{{\boldsymbol{H}}}}^{pe}=M{{\boldsymbol{N}}}^{{\boldsymbol{\sigma }}}\,{\dot{e}}^{p}/2(1+2{R}_{k}^{2})$, where the second order symmetric tensor ***N***^***σ***^ specifies the Tresca slip mechanism, while the rate of equivalent plastic strain is ${\dot{e}}^{p}$. The average Schmid factor *M* controls plastic hardening. The factor carries information on the misorientation of individual slip planes and is a function of plastic strain, temperature and is affected by dynamic resistance to flow *M* = *M*(*e*^*p*^,*A*_*D*_,*R*_*k*_).
3. The viscoplastic constitutive model couples the rate of equivalent plastic strain ${\dot{e}}^{p}$ with equivalent stress (maximum shear stress)${\dot{e}}^{p}={{\rm{\Lambda }}}_{0}\,{\dot{{\rm{\Lambda }}}}_{p}\cdot {({\sigma }_{eq}/{\sigma }_{0}^{p})}^{{n}_{p}}$. We assume that strength ${\sigma }_{0}^{p}={\sigma }_{0}{A}_{D}$ is sensitive to temperature and *σ*_0_ is a constant. The parameter Λ_0_ is used for the calibration of yield stress and the stress exponent *n*_*p*_ determines the elastic-plastic transition. The strain rate sensitivity is further tuned by the rate ${\dot{{\rm{\Lambda }}}}_{p}$.

The model is implemented to code Mathematica. The model calibration is based on experimental data collected for annealed OFHC copper (refs^4,9,17,18^). A complete list of parameters is presented in Methods.

**Figure 2:**
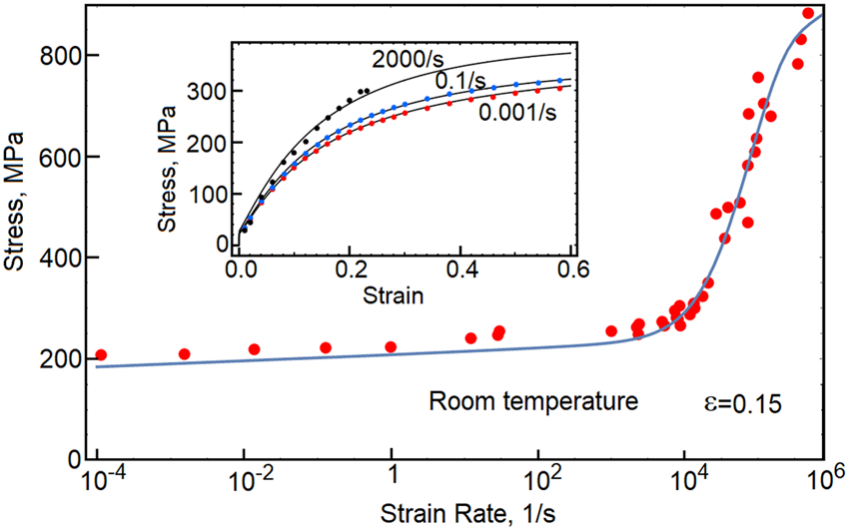
Stress at 15 percent strain is plotted as a function of strain rate. At strain rate above 10^3^/s, the dynamic strengthening affects the material responses. The model predictions are tested against experimental data (red dots) collected from refs^4,9,17–23^. Also, the embedded stress-strain plots reflect copper responses at strain rates 0.001/s, 0.1/s and 2000/s.

**Figure 3:**
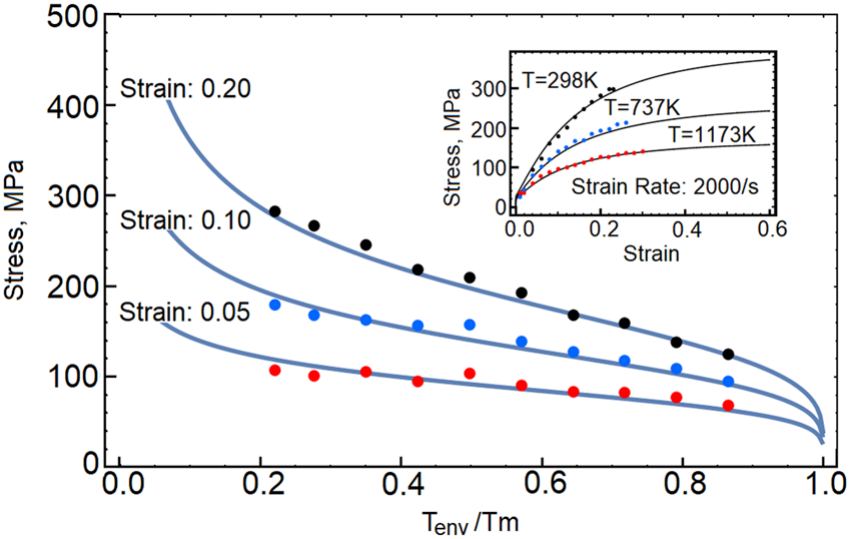
Stresses at 5, 10 and 15 percent strain are plotted as a function of temperature *T*_*env*_. The model predictions are compared with experimental data (red, blue and black points). Also, we include stress-strain plots at strain rate 2000/s, where the environment is at temperatures 298 K, 737 K and 1173 K. The experimental data is reported in ref.^9^.

**Figure 4:**
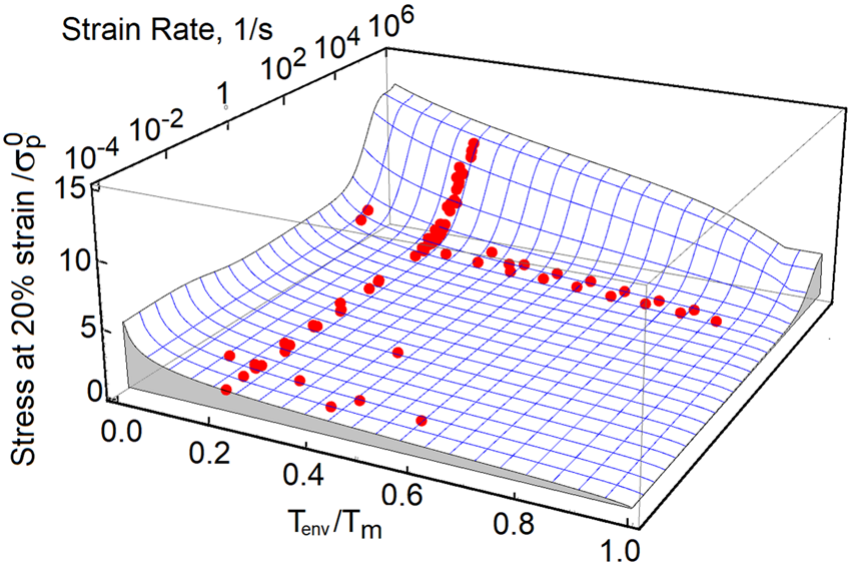
Stresses are collected at 20 percent strain and are plotted on blue mesh. The stresses are defined in terms of strain rate and temperature *T*_*env*_. Red dots represent experimental data obtained from refs^4,9,17–23^.

The strain rate sensitivity of OFHC copper is shown in Fig. 2. In this plot, stresses are collected at 15 percent strain. The red dots depict experimental data collected from refs^4,9,17,18^ and the model predictions are represented by solid lines. We also show three stress-strain responses at room temperature and at strain rates 0.001/s, 0.1/s and 2000/s. The black, blue and red dots represent experimental data^9^. The dynamic overstress affects the responses at strain rates above 10^4^/*s*, when deformation proceeds along the non-equilibrium trajectory marked in Fig. 1. The temperature sensitivity is shown in Fig. 3. As before, the black, blue and red dots represent experimental data reported in ref.^9^. Stresses are collected at strains 5, 10 and 20 percent, while the rate of strain is 2000/s. Also, we show three stress-strain responses calculated at the rate of 2000/s and temperatures 298 K, 773 K and 1173 K. The behavior is summarized in Fig. 4. In this plot, the predicted stresses at 20 percent strain are shown on the blue grid, where stresses are scaled by the yield stress (100 MPa). The strain rate is taken between 10^−4^/*s* and 10^6^/*s*, whereas temperature of the environment is in the range of 0.01*T*_*m*_ to 0.99*T*_*m*_. The experimental data includes additional results from refs^19–23^. The model properly captures the behavior within a broad range of temperatures between 77 K and 1173 K, while the strain rates are stretched over ten orders of magnitude. The magnitude of stress is affected by both of the resistance to flow *R*_*k*_ and the thermally activated reduction of the extrinsic resistance to flow *A*_*D*_. The two factors are in a direct competition. As a result, at melting point and high strain rates, the material retains measurable stresses. This phenomenon is observed in experiments^24^.

**Figure 5:**
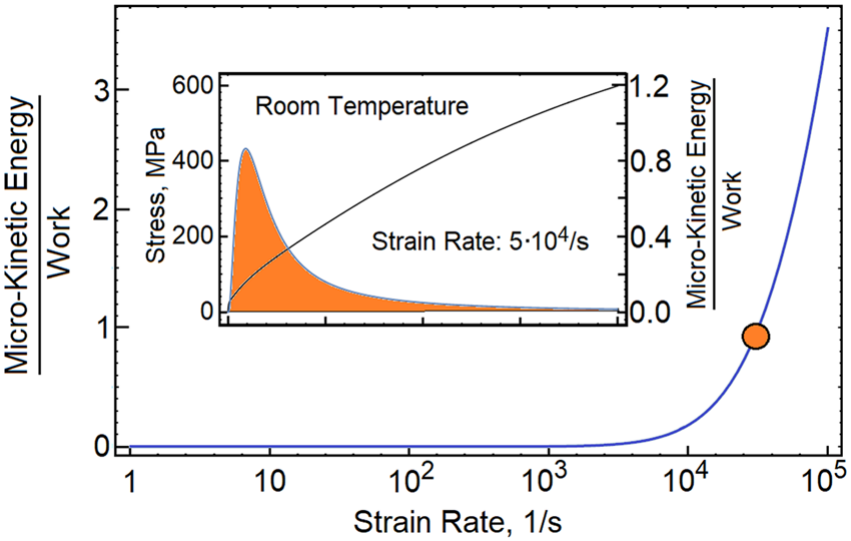
Peak magnitudes of micro-kinetic energy are plotted as a function of strain rate. At low strain rates, the energy is dissipated. At strain rates above 10^4^/s, the micro-kinetic energy becomes comparable with mechanical work.

Next, we plot the peak magnitudes of micro-kinetic energy as a function of strain rate, Fig. 5. Here, the maximum magnitude is scaled by mechanical work and is plotted as a function of the strain rate. The strongest manifestation of the non-equilibrium phenomena is observed during the elastic-plastic transition, where the dynamic excitations reach the maximum rate. Our predictions confirm the AE measurements^25,26^. Moreover, we note that the magnitude of AE counts is proportional to the strain rate. At strain rates above 10^5^/*s*, the excitations persist during the entire deformation process. We conjecture that there is an extreme loading, at which the excitations control the behavior.

In conclusion, we have shown that extreme dynamics create conditions, at which there is no time to properly convert energy to plastic work, and then, there is an energy excess that is partly stored in lattice and the rest of it is converted to micro-dynamic excitations. The dynamic storage is associated with the resistance to plastic flow. Furthermore, a part of energy is converted to micro-kinetic energy. The lifespan of the energy is short and is in the range of microseconds. At near melting temperature and high strain rates, we are able to reproduce the observed superheating. In our approach, we emphasize that the non-equilibrium phenomena are responsible for triggering the rapid multiplication of dislocations, which leads to the formation of fine dislocation structures. Thus, the fine dislocation structures result from but are not the root cause of the dynamic phenomena.

## Methods

The dynamic phenomena described in Results are studied with the use of our viscoplasticity model. The model has been used previously^27^ and is now modified to best serve the current purpose. As explained earlier, the model includes the descriptions of thermal activation, mechanisms-based plastic flow and modified power-law relation.

### Thermal activation

The thermal activation mechanism reflects the temperature-stimulated propensity of the material to overcome obstacles to plastic flow. In face-centered-cubic crystals, the activation mechanism affects plastic hardening^28,29^, while the dependence is not observed in bcc crystals. Here, we introduce a stochastic description of the activation processes and, later, the description is tuned to capture the behavior of fcc crystals. In this approach, we introduce the activation free energy *F*_*a*_ = *E*_*a*_−*S*_*a*_*T*. The energy consists of the activation energy *E*_*a*_ and there is the activation entropy *S*_*a*_, which describes the disorder that is predominantly caused by anharmonic effects of thermal expansion^30^. The disorder *S*_*a*_ makes the energy barriers weaker and the resistance to flow is reduced. The energy *E*_*a*_ and entropy *S*_*a*_ have been shown to be weakly dependent on temperature. We introduce a non-dimensional variable *ξ*_*a*_ = *F*_*a*_/*k*_*b*_*T*. The variable records the intensity of the thermally activated events. In here, *k*_*b*_ is Boltzmann constant and *T* is the actual temperature. The processes are stochastic with respect to *ξ*_*a*_. This means that the events of overcoming the flow obstacles are represented by a statistical distribution. As before, we make an assumption that it is the Weibull distribution. The Weibull distribution reflects the nature of the events, where the “weakest link” mechanism seems most appropriate when describing the phenomena. Consequently, the frequency of overcoming the flow obstacles is ${f}_{D}={k}_{a}{\xi }_{a}^{{k}_{a}-1}exp[-{\xi }_{a}^{{k}_{a}}]$. The material is expected to experience all the events that are available up to the temperature *T*. We introduce the transition temperature *T*_*c*_. At temperatures higher from *T*_*c*_, the material becomes increasingly softer and the flow mechanism undergoes a transition. The remaining resistance to the plastic flow *A*_*D*_ is estimated between the actual temperature and melting point ${A}_{D}={\int }_{{T}_{c}/{T}_{m}}^{{T}_{c}/T}{f}_{D}(s)ds$, where the temperature is scaled by the transition temperature *T*_*c*_. The variable *s* defines the temperature pathway *T*_*c*_/*T*. In here, the transition is associated with energy *k*_*b*_*T*_*c*_. We rewrite the activation energy ${E}_{a}={g}_{a}^{1/{k}_{a}}{k}_{b}{T}_{c}$ and introduce the energy multiplier *g*_*a*_. The activation entropy is calculated at melting point *F*_*a*_(*T*_*m*_) = 0 and we obtain *S*_*a*_ = *E*_*a*_/*T*_*m*_. The resistance to plastic flow becomes

10 $${A}_{D}(T)=1-exp[-{g}_{a}{(\frac{{T}_{c}}{T}-\frac{{T}_{c}}{{T}_{m}})}^{{k}_{a}}]\,.$$

Near melting point, where *A*_*D*_(*T*_*m*_) → 0, the plastic flow is free of resistance. On the other hand, the material exhibits the strongest resistance at low temperatures, where the factor *A*_*D*_ is only slightly smaller than one. Note that the dynamic constraints *R*_*k*_ and the factor *A*_*D*_ are constructed in the same way. In fcc crystals, the factor *A*_*D*_ affects both of the material strength and plastic hardening. In bcc metals, the thermal activation has been decoupled from plastic hardening^9,28^.

### Mechanisms-based plastic flow

We assume that the macroscopically observed plastic deformation follows the Tresca mechanism. In here, the plane of maximum shear is constructed on two orthogonal unit vectors ***n***^*σ*^and ***s***^*σ*^so the flow tensor is ${{\boldsymbol{N}}}^{{\boldsymbol{\sigma }}}=({{\boldsymbol{n}}}^{\sigma }\otimes {{\boldsymbol{s}}}^{\sigma }+{{\boldsymbol{s}}}^{\sigma }\otimes {{\boldsymbol{n}}}^{\sigma })$. We express the tensor ***N***^***σ***^ in terms of stress, where the representation is expressed in the general form ***N***^*σ*^ = *α*_0_1 + *α*_1_***S*** + *α*_2_***S·S*** and stress deviator is ***S*** = ***σ***−**1***tr***σ**/3. The three variables *α*_0_, *α*_1_ and *α*_2_ are functions of stress invariants. The representation must reproduce invariants of the original dyadic product $({{\boldsymbol{n}}}^{\sigma }\otimes {{\boldsymbol{s}}}^{\sigma }+{{\boldsymbol{s}}}^{\sigma }\otimes {{\boldsymbol{n}}}^{\sigma })$, thus *tr****N***^***σ***^ = 0, *tr*(***N***^***σ***^)^2^ = 2 and ***N***^***σ***^ = (***N***^***σ***^)^3^. We obtain three solutions and select the one that corresponds to the maximum shear mechanism^31^, hence

11 $${{\boldsymbol{N}}}^{{\boldsymbol{\sigma }}}=\frac{2\,\cos \,\frac{\phi }{3}}{\sqrt{3}\,\cos \,\phi }(1-\frac{3}{2{J}_{2}}{{\boldsymbol{S}}}^{2})+\frac{\cos \,\frac{2\phi }{3}}{\sqrt{{J}_{2}}\,\cos \,\phi }{\boldsymbol{S}}.$$

The angle is $\phi ={\sin }^{-1}({J}_{3}\sqrt{27/4\,}/{J}_{2}^{3}\,)$ and the second and third invariants of stress deviator are *J*_2_ and *J*_3_, respectively.

The macroscopic flow mechanism ***N***^***σ***^ summarizes local slip events in the stressed material^31,32^. The rates of local plastic strains are collected from individual slip systems with orientations distributed around the plane of the maximum shear stress. Each such *θ*-active system is constructed on unit vectors ***n***^*θ*^ and ***s***^*θ*^, where the *θ*-flow tensor is ${{\boldsymbol{N}}}^{\theta }=({{\boldsymbol{n}}}^{\theta }\otimes {{\boldsymbol{s}}}^{\theta }+{{\boldsymbol{s}}}^{\theta }\otimes {{\boldsymbol{n}}}^{\theta })$ and *tr****N***^*θ*^ = 0. For simplicity, the *θ*-planes are coplanar with the plane of maximum shear stress. As plastic deformation advances, the plastic slip undergoes rearrangements such that ${\dot{{\boldsymbol{n}}}}^{\theta }=\dot{\theta }{{\boldsymbol{s}}}^{\theta }$ and ${\dot{{\boldsymbol{s}}}}^{\theta }=-\dot{\theta }{{\boldsymbol{n}}}^{\theta }$. We assume that the macroscopic behavior is isotropic. This means that grains in the polycrystal do not exhibit preferential orientations and material points (in the sense of continuum) carry information gathered from a large number of such grains. Consequently, we assume that *θ*-systems are continuously distributed and the distribution *f*_*θ*_(*θ*) of *θ*-slippages is taken between ±*π*/4. In plastically isotropic metals, the distribution is sufficiently general when ${f}_{\theta }(\theta )={f}_{\theta }^{0}{\cos }^{1/r}2\theta$and ${f}_{\theta }^{0}=2{\rm{\Gamma }}(1+1/2r)/\sqrt{\pi }{\rm{\Gamma }}(1/2+1/2r)$. In this way, we have ${\int }_{-\pi /4}^{\pi /4}{f}_{\theta }(\theta )d\theta =1$ and Γ is gamma function. The exponent *r* determines shape of the distribution. As plastic deformation increases, the slip interactions broaden the distribution and create conditions for plastic hardening. At macroscale, the rate of macroscopic plastic strain ${\dot{{\boldsymbol{H}}}}^{pe}$ includes the contributions from all active systems and is sensitive to the microstructural rearrangements^33^, therefore

12 $${\dot{{\boldsymbol{H}}}}^{pe}=\frac{1}{2(1+2{R}_{k}^{2})}{\int }_{-\pi /4}^{\pi /4}[\partial ({{\boldsymbol{N}}}^{\theta }{e}^{\theta })/\partial t]d\theta .$$

The equivalent plastic strain *e*^*θ*^ specifies the *θ*-slip magnitude. The rate of *θ*-slip is scaled by the function *f*_*θ*_(*θ*) such that ${\dot{e}}^{\theta }={f}_{\theta }(\theta ){\dot{e}}^{p}$ and the macroscopic equivalent plastic strain is $\,{\dot{e}}^{p}$. The slip reorientations are triggered by local slip events and, therefore, we have $\dot{\theta }=(\partial \theta /\partial {e}^{\theta }){\dot{e}}^{\theta }$. Hence, the rate of plastic strain is${\dot{{\boldsymbol{H}}}}^{pe}=[{\int }_{-\pi /4}^{\pi /4}(\,{{\boldsymbol{N}}}^{\theta }-{{\boldsymbol{N}}}^{R})$ ${f}_{\theta }(\theta ){\dot{e}}^{p}d\theta ]/2(1+2{R}_{k}^{2})$, whereas the evolution is given in ${{\boldsymbol{N}}}^{R}=2({{\boldsymbol{n}}}^{\theta }\otimes {{\boldsymbol{n}}}^{\theta }-{{\boldsymbol{s}}}^{\theta }\otimes {{\boldsymbol{s}}}^{\theta }){e}_{m}^{\theta }(\partial \theta /\partial {e}_{m}^{\theta })$. The reorientations widen the distribution *f*_*θ*_(*θ*) and, effectively, reduce the rate of plastic flow. The slip systems that are closely aligned with the direction of maximum shear stress (*θ* = 0) do not rotate^33^. The strongest propensity for reorientations is at the angles ±*π*/4. The mechanism is described by the relation ${e}^{\theta }(\partial \theta /\partial {e}^{\theta })={\alpha }_{p}\,\sin \,2\theta$, where the parameter *α*_*p*_ controls the rate of the reorganizations. At *θ* = ±*π*/4, shear stress is equal to zero and, therefore, the slip rearrangements are most active somewhere between *θ* = 0 and *θ* = ±*π*/4. Next, we express ***n***^*θ*^ and ***s***^*θ*^ in terms of the macroscopic mechanism (***n***^*σ*^ and ***s***^*σ*^**)** such that ${{\boldsymbol{n}}}^{\theta }={{\boldsymbol{n}}}^{\sigma }\,\cos \,\theta +{{\boldsymbol{s}}}^{\sigma }\,\sin \,\theta$ and ${{\boldsymbol{s}}}^{\theta }={{\boldsymbol{s}}}^{\sigma }\,\cos \,\theta -{{\boldsymbol{n}}}^{\sigma }\,\sin \,\theta$. In the *R*_*k*_-constrained configuration, the rate of flow becomes

13 $${\dot{{\boldsymbol{H}}}}^{pe}=\frac{M}{2(1+2{R}_{k}^{2})}{{\boldsymbol{N}}}^{{\boldsymbol{\sigma }}}{\dot{e}}^{p},$$

where the average Schmid factor *M* = *M*_*s*_−*M*_*r*_ is responsible for plastic hardening. The factor reflects the current configuration of slip systems *M*_*s*_ = Γ^2^(1 + 1/2*r*)/[Γ(1/2 + 1/2*r*)Γ(3/2 + 1/2*r*)] and includes the contribution of the slip rearrangements *M*_*r*_ = *α*_*p*_2*r*/(1 + 2*r*). The exponent *r* is a function of plastic strain and temperature and is also scaled by the dynamic constraints. We propose a simple relation $\,r={A}_{D}\,{r}_{0}\,(1+\varsigma )/(1+2\,{R}_{k}^{2})$, where plastic strain $\,\varsigma ={e}^{p}/{e}_{0}^{p}$ is scaled by a constant $\,{e}_{0}^{p}$.

#### Power-law relations

The rate of plastic work ${\dot{W}}^{p}=({\boldsymbol{\sigma }}:{{\boldsymbol{N}}}^{{\boldsymbol{\sigma }}}/2)[M{\dot{e}}^{p}/(1+2{R}_{k}^{2})]$ is expressed in terms of equivalent stress *σ*_*eq*_ = ***σ***:***N***^***σ***^/2 and the equivalent strain rate is ${\dot{e}}^{pe}=M{\dot{e}}^{p}/(1+2{R}_{k}^{2})$. We introduce the rate of effective strain

14 $${\dot{e}}^{pe}={\dot{{\rm{\Lambda }}}}_{p}\frac{M{{\rm{\Lambda }}}_{0}}{1+2{R}_{k}^{2}}{({\sigma }_{eq}/{\sigma }_{0}^{p})}^{{n}_{p}}.$$

Strength ${\sigma }_{0}^{p}$ is sensitive to temperature and is ${\sigma }_{0}^{p}={\sigma }_{0}{A}_{D}$, while the athermal strength *σ*_0_ is a pre-determined constant. The yield stress is adjusted with the use of the constant Λ_0_. We correct the power-law relation (14) and introduce a rate-sensitive factor ${\dot{{\rm{\Lambda }}}}_{p}={\dot{e}}_{N}^{0}{[{\dot{e}}_{N}^{t}/{\dot{e}}_{N}^{0}+{({\dot{e}}_{D}/{\dot{e}}_{N}^{0})}^{1/(1-{A}_{D})}]}^{{\omega }_{p}}$, where ${\dot{e}}_{N}^{t}={({\dot{{\boldsymbol{H}}}}^{t}:{\dot{{\boldsymbol{H}}}}^{t}/2)}^{1/2}$ and the reference rate ${\dot{e}}_{N}^{0}=1/s$ is given in seconds. The term ${({\dot{e}}_{D}/{\dot{e}}_{N}^{0})}^{1/(1-{A}_{D})}$ describes the contribution of diffusional flow, in which the parameter $\,{\dot{e}}_{D}$ is a constant and is in the range of 10^−6^/*s*. At very low strain rates, the term $({\dot{e}}_{D}/{\dot{e}}_{N}^{0})$ becomes increasingly relevant and the strain rate dependence converges to ${\dot{e}}^{pe}\propto {({\sigma }_{eq}/{\sigma }_{0}^{p})}^{{n}_{p}}$. When temperature is high (*T* ≥ *T*_*c*_) and strain rates are below 10^−5^/*s*, the term ${({\dot{e}}_{D}/{\dot{e}}_{N}^{0})}^{1/1-{A}_{D}}$ controls diffusional flow^34^. At the initial stage of plastic deformation, the rate of elastic strain dominates $({\dot{{\boldsymbol{H}}}}^{e}\to {\dot{{\boldsymbol{H}}}}^{t})$ and, therefore, we have ${\dot{e}}^{pe}\propto {({\sigma }_{eq}^{p}/{\sigma }_{0}^{p})}^{{n}_{p}}$. When the plastic strain rate controls the deformation process $({\dot{{\boldsymbol{H}}}}^{pe}\to {\dot{{\boldsymbol{H}}}}^{t})$, the strain rate sensitivity is ${\dot{e}}^{pe}\propto {({\sigma }_{eq}^{p}/{\sigma }_{0}^{p})}^{{n}_{p}/(1-{\omega }_{p})}$. Thus, the material experiences a smooth elastic-plastic transition.

### Parameters

The experimental results for annealed OFHC copper are obtained from refs^4,9,17,18^. The parameters are collected in five groups. The first order calibration is completed independently in each group and fine-tuning of the parameters followed. The parameters are listed below

- Basic constants: $${\mu }_{0}=45GPa;{B}_{0}=120GPa;{C}_{p}=386J/kg\cdot K;\rho =8960kg/{m}^{3}$$
- Thermal activation: *k*_*a*_ = 0.11;*g*_*a*_ = 1.135;*T*_*m*_ = 1356;*T*_*c*_ = 0.65*T*_*m*_
- Dynamic excitations: *τ*_0_ = 10^−7^/*s*;*G*_*R*_ = 3*MJ*/*m*^3^;*k*_*R*_ = 0.33
- Average Schmid factor: ${r}_{0}=0.32;{e}_{0}^{p}=0.0125;{\alpha }_{p}=2.0/\pi$
- Viscoplasticity: $${\sigma }_{0}=100MPa;{{\rm{\Lambda }}}_{0}=7.3;{n}_{p}=0.5;{\omega }_{p}=0.993;{\dot{e}}_{D}^{0}=1\cdot \,{10}^{-6}/s$$

Elastic properties are sensitive to temperature^35^, where the best fit to the experimental data gives *μ* = *μ*_0_(1−0.437*T*/*T*_*m*_) and *B* = *B*_0_(1−0.286*T*/*T*_*m*_). Also, we make a correction for the adiabatic heating. The rule of thumb is that the isothermal-to-adiabatic transition occurs at strain rates ${\dot{e}}_{N}^{t}={({\dot{{\boldsymbol{H}}}}^{t}:{\dot{{\boldsymbol{H}}}}^{t}/2)}^{1/2}$ between 0.001/s and 10/s. The relation ${\xi }_{T}=0.9\,[1+tanh(lo{g}_{10}{\dot{e}}_{N}^{t}+1)]/2$ closely reproduces the rule. The material is tested at temperature *T*_*env*_, but the true temperature is *T* = *T*_*env*_ + Δ*T*, where ${\rm{\Delta }}\dot{T}=[{\xi }_{T}{\dot{W}}^{p}+(1+2{R}_{k}^{2})\,{\dot{D}}_{k}]/(\rho \cdot {C}_{p})$.

### Availability of materials and data

All data analyzed during this study are included in this published article.
